# Zombies Never Die: The Double Life Bub1 Lives in Mitosis

**DOI:** 10.3389/fcell.2022.870745

**Published:** 2022-05-13

**Authors:** Yuqing Zhang, Chunlin Song, Lei Wang, Hongfei Jiang, Yujing Zhai, Ying Wang, Jing Fang, Gang Zhang

**Affiliations:** ^1^ The Cancer Institute, The Affiliated Hospital of Qingdao University, Qingdao, China; ^2^ School of Public Health, Qingdao University, Qingdao, China

**Keywords:** mitosis, spindle assembly checkpoint, kinetochore, Bub1, chromosome alignment

## Abstract

When eukaryotic cells enter mitosis, dispersed chromosomes move to the cell center along microtubules to form a metaphase plate which facilitates the accurate chromosome segregation. Meanwhile, kinetochores not stably attached by microtubules activate the spindle assembly checkpoint and generate a wait signal to delay the initiation of anaphase. These events are highly coordinated. Disruption of the coordination will cause severe problems like chromosome gain or loss. Bub1, a conserved serine/threonine kinase, plays important roles in mitosis. After extensive studies in the last three decades, the role of Bub1 on checkpoint has achieved a comprehensive understanding; its role on chromosome alignment also starts to emerge. In this review, we summarize the latest development of Bub1 on supporting the two mitotic events. The essentiality of Bub1 in higher eukaryotic cells is also discussed. At the end, some undissolved questions are raised for future study.

## Introduction

Genetic materials need to be accurately passed to the next generation which ensures the continuation of life. In eukaryotes, the chromosomes carrying the genetic information are replicated first and then equally segregated into daughter cells during mitosis. To secure the equal segregation, each pair of replicated chromatids need to be correctly attached by the spindle microtubules on the kinetochores, which is a proteinaceous structure built on centromeric chromosome. Afterwards, the physical connection between the sister chromatids is cleaved and each chromatid move towards the spindle pole driven by the shortening of microtubules attached to kinetochore. Finally, an actomyosin-based contractile ring forms at the cortex between the spindle poles and constricts to divide the cell into two. Due to the existence of multiple chromosomes in eukaryotic cells, the generation of stable attachment between kinetochores of all the chromosomes and spindle microtubules needs a certain time. Only when all the kinetochores have been properly attached by microtubules, chromosome segregation starts. Otherwise, a premature segregation in the presence of unattached chromosomes will cause gain or loss of chromosomes in daughter cells which could result in severe problems like cell death, miscarriage, developmental defects or tumorigenesis. To fulfill the temporal requirement, eukaryotic cells have evolved a monitoring mechanism called the spindle assembly checkpoint (SAC). Unattached kinetochores activate SAC which catalyzes the formation a protein complex termed mitotic checkpoint complex (MCC). MCC is composed by three checkpoint proteins BubR1, Bub3, Mad2 and cell division cycle 20 (Cdc20), which is a coactivator of E3 ubiquitin ligase Anaphase-Promoting Complex or Cyclosome (APC/C). Cdc20 activated APC/C promotes the transition from metaphase to anaphase by degrading a few key mitotic regulators. MCC binds and inhibits APC/C, thus delays the onset of anaphase until the proper kinetochore-microtubule attachments are established. Once the stable attachment is generated, kinetochores stop producing MCC and existing MCC gets disassembled. Only now, the cells could enter anaphase (reviewed in Musacchio, 2015; Kapanidou et al., 2017; Maiato et al., 2017; Lara-Gonzalez et al., 2021a).

Budding Uninhibited by Benzimidazoles 1 (*BUB1*) was first identified from the pioneer screening for mitotic checkpoint genes (Hoyt et al., 1991; Li et al., 1991). Bub1 protein was soon characterized as a serine/threonine kinase by the ability to phosphorylate itself and associated Bub3 protein (Roberts et al., 1994). Besides its role on SAC, early study also discovered another crucial function of Bub1 in maintaining genome stability (Bernard et al., 1998). Since then, numerous studies have greatly advanced our knowledge of the functions Bub1 plays in mitosis. Among the multiple functions, the role of Bub1 in activating the spindle assembly checkpoint is the most studied. Now it is clear that Bub1 is required for the full activation of the checkpoint through multiple mechanisms though the extent of requirement is context-dependent. How does Bub1 promote chromosome alignment is not fully understood. Recent studies have shed some lights on this functionality. Besides these two roles, Bub1 is also involved in chromatid cohesion protection through recruiting Sgo1/PP2A and this role seems more essential in meiosis than in mitosis (Kitajima et al., 2004; Tang et al., 2004; Kitajima et al., 2005; Tang et al., 2006; Perera et al., 2007; Schliekelman et al., 2009; Miyazaki et al., 2017; Yi et al., 2019; Carvalhal et al., 2022). Bub1 may be required for telomere replication (Li et al., 2018) and activation of DNA damage response (Hein et al., 2009). Due to the size limit of this review, we only discuss the latest development of Bub1 on activating SAC and promoting chromosome alignment. We try to dissect the functions through examining individual interactions along with the kinase activity. At the end we discuss the essentiality of Bub1 in human cells and the questions remain to be answered.

## The Role of Bub1 in the Spindle Assembly Checkpoint

The molecular mechanism of the SAC has been thoroughly reviewed and we refer the reader to excellent reviews on the topic (Musacchio, 2015; Lara-Gonzalez et al., 2021a). Here we focus on the checkpoint protein Bub1 with an emphasis on recent discoveries.

**FIGURE 1:**
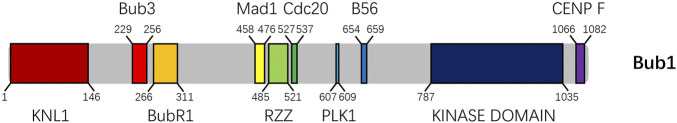
Schematic of the domains on human Bub1 protein. From left to right, the domains interacting with KNL1, Bub3, BubR1, Mad1, RZZ, Cdc20, Plk1, B56 (putative) and CENP-F are marked with reported boundaries. The kinase domain is also shown. The numbers below or above the schematic indicate the start and end of the boundaries.

Unoccupied kinetochores activate SAC. In line with this, all the SAC proteins associate dynamically with the outer kinetochores in early mitosis. Understanding the molecular mechanisms of the kinetochore localization is key to comprehend the SAC activation and silencing. Bub1 was speculated to be one of the first checkpoint components on kinetochores (Jablonski et al., 1998). The kinetochore localization of Bub1 was found to depend on its association with Bub3. Bub1 makes a stable complex with Bub3 through a domain in the N-terminal region of Bub1 (Taylor et al., 1998). Subsequent studies found that KNL1 was required for Bub1 kinetochore localization and this was suggested to be driven by an interaction between a KI motif in KNL1 and the N terminal tetratricho-peptide repeat (TPR) domain of Bub1 (Kiyomitsu et al., 2007; Kiyomitsu et al., 2011). However, this model did not readily explain the requirement for Bub3 and subsequent work questioned the relevance of the KI-TPR domain interaction for Bub1 localization (Krenn et al., 2012). Further studies showed that a phosphorylation-dependent interaction between KNL1 and Bub1-Bub3 was required for Bub1 kinetochore localization. The checkpoint kinase Mps1 phosphorylates a repetitive motif composed of the consensus sequence [M/I] [E/D/N] [I/L/M] [S/T] (named MELT motif) on KNL1. The phosphorylated MELT motifs provide docking sites for Bub1/Bub3 *via* a direct interaction with Bub1-Bub3 (London et al., 2012; Shepperd et al., 2012; Yamagishi et al., 2012; Primorac et al., 2013; Zhang et al., 2014; Vleugel et al., 2015b; Roy et al., 2020). This interaction is tightly regulated by protein phosphatases PP2A/B56 and PP1 to ensure ontime SAC silencing (London et al., 2012; Espert et al., 2014; Nijenhuis et al., 2014; Zhang et al., 2014; Roy et al., 2019; Braga et al., 2020). Since the establishment of how Bub1 is recruited to kinetochores, the hierarchy of the SAC has become clear. Now it is known that Bub1 is a major hub for assembly of the SAC machinery through direct interaction with BubR1, Mad1-Mad2, Cdc20, as well as interaction with the RZZ complex. These interactions and their function in the SAC are discussed in an order from the N-terminus to the C-terminus of Bub1 in the subsequent sections (Figure 1).

### Bub1-BubR1 Interaction Contributes to Spindle Assembly Checkpoint Silencing

BubR1 is the key component of MCC which inhibits APC/C directly by blocking the access of the substrates. Interestingly, *BUBR1* and *BUB1* are paralog genes and the gene products share highly structural similarity. Sub-functionalization during evolution confers the scaffold functionalities to Bub1 and MCC functionalities to BubR1 (Kops et al., 2020). Early studies suggested an interaction between Bub1 and BubR1 (Millband and Hardwick, 2002; Johnson et al., 2004; Rischitor et al., 2007; Kiyomitsu et al., 2011). The interaction was later revealed to be through direct binding of the regions adjacent to the Bub3 binding domain on each protein (Overlack et al., 2015; Zhang et al., 2015). This interaction is required for efficient kinetochore localization of BubR1. The reason BubR1-Bub3 does not efficiently localize to kinetochores is because there are direct contacts between Bub1 and the phosphorylated MELT motifs. This direct contact is mediated by a unique loop in Bub1 that increases the binding affinity with phosphorylated MELT motifs (Overlack et al., 2015). Disrupting the direct interaction between Bub1-BubR1 did not reduce but increased the checkpoint strength which likely reflects the role of checkpoint silencing by the protein phosphatase PP2A-B56 associated with BubR1 (Espert et al., 2014; Zhang et al., 2015; Zhang et al., 2016; Qian et al., 2017).

One interesting question raised here is whether the kinetochore localized BubR1 is important for the checkpoint activity. Based on the fact that low amounts of BubR1 remain on kinetochores following complete removal of Bub1, we proposed a model that two populations of BubR1 exist on kinetochores. The Bub1-dependent BubR1 contributes to the chromosome alignment and the Bub1-independent BubR1 is important for the checkpoint activation (Zhang et al., 2016). The existence of a Bub1-independent pool of BubR1 has been observed on kinetochores in many studies (Overlack et al., 2015; Vleugel et al., 2015a; Zhang et al., 2016; Raaijmakers and Medema, 2019; Chen et al., 2021). FRAP analysis also identified a slow population and a fast population of BubR1 on unattached kinetochores. The t_1/2_ of the slow BubR1 is very close to the t_1/2_ of Bub1 (Howell et al., 2004). Besides, Bub1 alone does not saturate the phosphorylated MELT motifs which leaves spaces for the binding of BubR1-Bub3 to the vacant MELT motifs (Overlack et al., 2015). In fission yeast BubR1 (Mad3) expresses three to four times to Bub1 which is also likely the case in human cells (Heinrich et al., 2013). All these aspects indicate a direct binding of BubR1-Bub3 with phosphorylated MELT motif could co-exist with the heterodimerization of BubR1-Bub3 with Bub1-Bub3. As discussed below, this more dynamic pool of BubR1-Bub3 could interact with newly formed Mad2-Cdc20 complex in close proximity and form MCC which leaves kinetochores to inhibit the APC/C.

### Bub1-Mad1 Interaction is Critical for Spindle Assembly Checkpoint Activation

How unattached kinetochores catalyze the generation of MCC is the key question in understanding SAC signaling. The prevailing model is centered on the Mad1:Mad2 complex as a key catalyst for loading Mad2 onto Cdc20. Mad1 contains a long predicted coiled-coil in the N-terminal region and a RWD (RING finger-, WD-repeat-, and DEAD-like proteins) domain at the C-terminus. Dimerized Mad1 forms a stable tetramer with two molecules of Mad2. There are two conformations of the Mad2 protein, open Mad2 (O-Mad2) and closed Mad2 (C-Mad2), and Mad2 exists in the closed form when bound to its ligands Mad1 and Cdc20. The kinetochore localized Mad1:C-Mad2 recruits O-Mad2 from the cytoplasm through heterodimerization and converts it into an empty C-Mad2 intermediate which rapidly binds Cdc20 to form the Mad2-Cdc20 complex (Luo et al., 2002; Sironi et al., 2002; Shah et al., 2004; De Antoni et al., 2005; Mapelli et al., 2006; Mapelli et al., 2007; Luo and Yu, 2008; Yang et al., 2008; Kim et al., 2012; Hara et al., 2015). Afterwards, BubR1-Bub3 binds Mad2-Cdc20 to form the MCC complex which can diffuse and inhibit the APC/C (Kulukian et al., 2009). Obviously, discovering the mechanism of Mad1 kinetochore localization is important to test the above model and infer further mechanistic insight into SAC signaling. In mammalian cells, multiple proteins have been proposed as the kinetochore receptors for Mad1 but work is converging on Bub1 and the RZZ complex as the relevant receptors (reviewed in Luo et al., 2018). Recent studies confirmed these two distinct but integrated pathways responsible for Mad1 kinetochore localization and functioning (Silió et al., 2015; Rodriguez-Rodriguez et al., 2018; Zhang et al., 2019). The two pathways will be discussed separately in the following sections.

**FIGURE 2:**
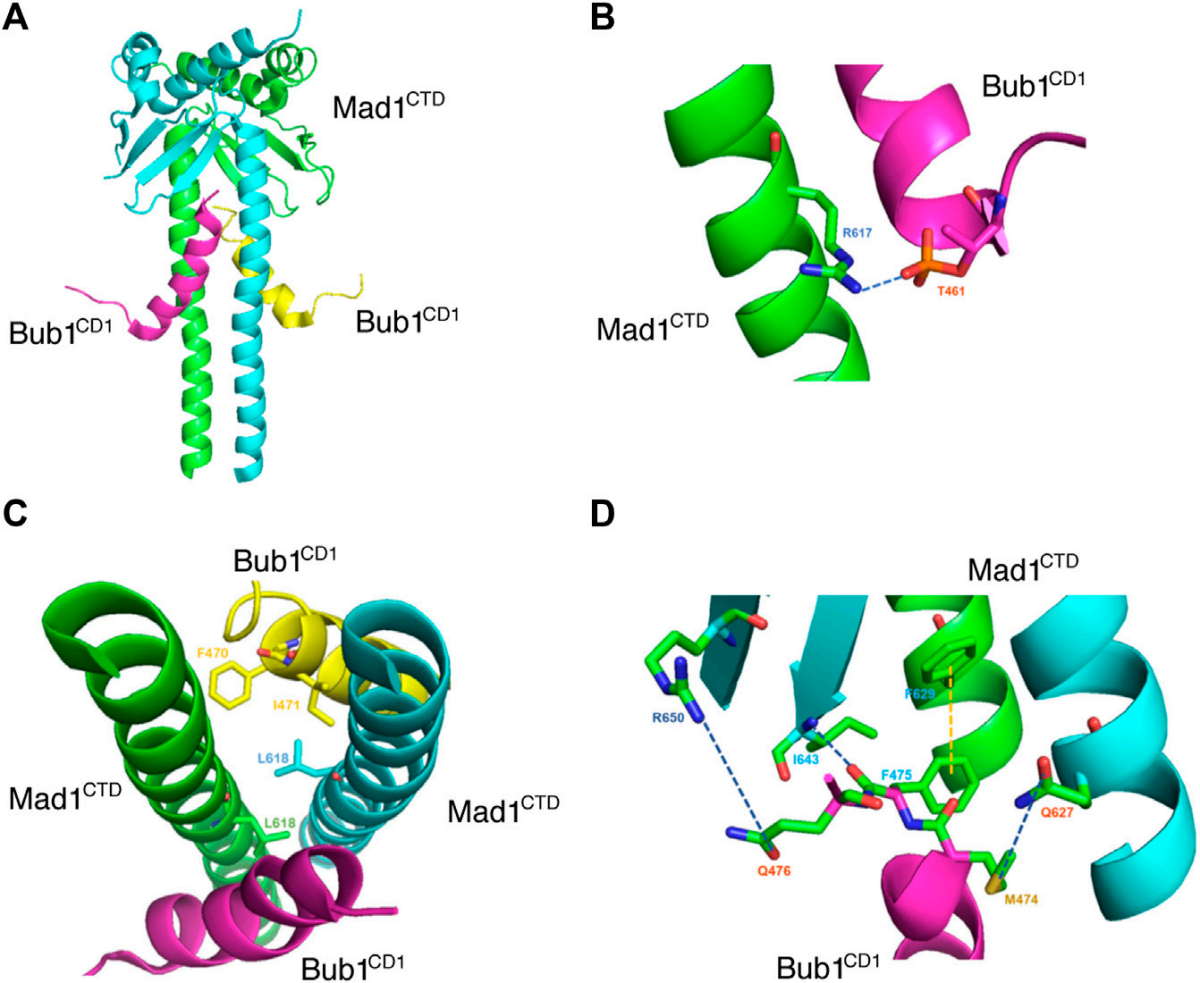
Crystal structure of Mad1CTD-Bub1CD1 shows multiple direct interactions. **(A)** Crystal structure of Mad1CTD dimer (green/light blue) bound to two Bub1CD1 molecules (purple/yellow) from PDB: 7B1F (Fischer et al., 2021). **(B)** Close-up view of the interaction between Mad1 R617 with Bub1 pT461. The dark blue dashes indicate the hydrogen bonding. **(C)** Top view of the hydrophobic pocket formed by Mad1 L618 and Bub1 F470 and I471. **(D)** Close-up view of the interactions between Mad1 R650 and Bub1 Q476, Mad1 I643 and Bub1 F475, Mad1 F629 and Bub1 F475, Mad1 Q627 and Bub1 M474. The dark blue dashes indicate hydrogen bonding and the yellow dashes indicate π–π stacking. Pymol was used to visualize the structure and interactions between residues.

The Bub1-Mad1 interaction was originally reported to be crucial for checkpoint activation in budding yeast (Brady and Hardwick, 2000). A conserved Arg-Leu-Lys sequence (RLK motif) on Mad1 was found to be required for the interaction while the corresponding motif on Bub1 was only revealed recently. In fission yeast, the middle region of Bub1 bound directly to Mad1 C-terminal region and the interaction required that Bub1 was phosphorylated by Mps1 on a conserved site. The binding was critical for Mad1 kinetochore localization and SAC activation (London and Biggins, 2014; Mora-Santos et al., 2016). In mammalian cells, the region on Bub1 involved in the interaction was narrowed down to a conserved domain 1 (CD1) (Klebig et al., 2009; Ji et al., 2017; Zhang et al., 2017; Qian et al., 2017). Within the CD1 domain, two phosphorylation happens sequentially at S459 and T461. The phosphorylation of S459 by CDK1 primes the phosphorylation of T461 by Mps1 which then mediates the interaction with Mad1 RLK motif. However, even with double phosphorylation, the affinity between Bub1 CD1 peptide and recombinant Mad1 C-terminal protein is relatively low which explains the difficulty to detect the Bub1-Mad1 interaction in human cells (Ji et al., 2017; Zhang et al., 2017). Subsequent dephosphorylation of the two sites by PP2A-B56 makes the detection even more difficult (Qian et al., 2017). An interesting feature of CD1 domain is the presence of a theoretical alpha helix starting at T464 following the two phosphorylation sites. Mutagenesis of residues within the helix region abolishes the Bub1-Mad1 interaction and the checkpoint indicating more complicated interactions besides the electrostatic interaction (Zhang et al., 2017). In a recent structure study, Fischer et al. (2021) found pT461, but not pS459 formed a direct contact with R617 on Mad1 (Figures 2A,B). Interestingly, the contact between pT461 and R617 caused conformational changes on Bub1 CD1 domain into an alpha-helix starting from pT461 instead of the predicted T464. A simultaneous conformation rearrangement also happened on Mad1 after bound to phosphorylated Bub1 which further enhanced the interaction with the formation of a hydrophobic pocket and multiple contacts between the CD1 alpha-helix and Mad1 homodimer (Fischer et al., 2021; Figures 2C,D). The structural study is fully consistent to the previous functional analysis (Ji et al., 2017; Zhang et al., 2017; Fischer et al., 2021). Besides CDK1 and Mps1, Aurora B was recently reported to promote Bub1-Mad1 interaction when it was induced to dimerize with Bub1 (Roy et al., 2022). It will be interesting to test the model under physiological conditions in future work.

**FIGURE 3:**
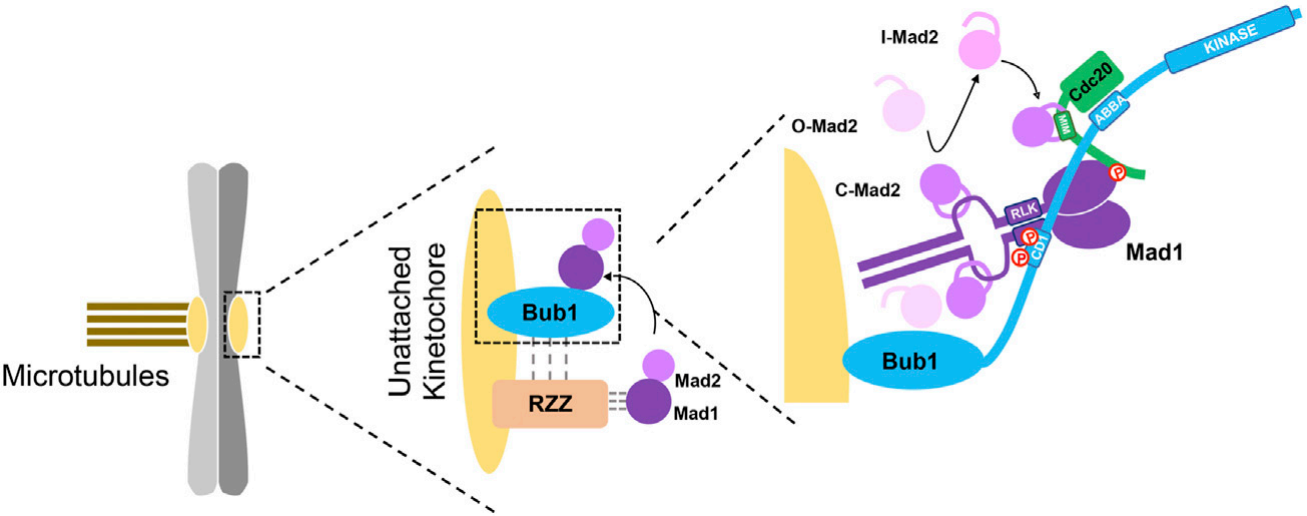
Bub1 activates SAC via interactions with multiple checkpoint proteins. The cartoon describes the interactions of Bub1 with Mad1, RZZ and Cdc20 in activating SAC signaling on unattached kinetochores. The left part shows a pair of chromatids with one kinetochore attached by microtubules and the other one unattached. The middle part shows recruiting Mad1/Mad2 onto kinetochores through two distinct and integrated pathways, Bub1-Mad1 and RZZ-Mad1. The dashed lines indicates dependency of RZZ on Bub1 and Mad1 on RZZ whereas the physical interactions have not been confirmed. RZZ binding with Mad1 promotes the binding between Bub1 and Mad1, which further catalyzes the formation of Mad2-Cdc20 complex as shown in the right part. Simultaneous interaction of Bub1 with Mad1-Mad2 and Cdc20 brings Mad2 close to Cdc20. The phosphorylation-mediated binding of the N-terminal Cdc20 with the C-terminal Mad1 exposes the Mad2 interacting motif on Cdc20 and facilitates the formation of Mad2-Cdc20 complex. The minor role of the kinase activity of Bub1 in SAC activation is not described here. Adapted from Lara-Gonzalez et al. (2021b).

Adjacent to the CD1 domain is a Cdc20 binding motif (ABBA motif or Phe box) which directly interacts with a binding pocket on Cdc20 WD 40 repeats and recruits Cdc20 onto kinetochores (Di Fiore et al., 2015; Diaz-Martinez et al., 2015). Interestingly, a basic patch on the N-terminal region of Cdc20 is able to bind the C-terminus of Mad1 after Mad1 is phosphorylated by Mps1. The binding relieves the intramolecular interaction within Cdc20, thus exposes the Mad2 interacting motif (MIM) to the ligand-free intermediate C-Mad2. Therefore, the simultaneously binding of Cdc20 and Mad1 with Bub1 facilitates the initial assembly of Mad2-Cdc20 complex (Ji et al., 2017; Faesen et al., 2017; Piano et al., 2021; Lara-Gonzalez et al., 2021b; Figure 3). The remaining question is how intermediate C-Mad2 meet Cdc20. The newly resolved Bub1-Mad1 structure positions Bub1-bound Cdc20 in between the Mad2 binding domain and the C-terminus of Mad1. So intermediate C-Mad2 may leave Mad1-Mad2 molecules and capture the close-by exposed MIM on Cdc20. There are evidences showing Cdc20 is one of the kinetochore receptors of C-Mad2 but not vice versa (Lischetti, et al., 2014; Zhang et al., 2018; Figure 3). After the initial formation, Mad2-Cdc20 complex will rapidly bind BubR1/Bub3 to form the final MCC to regulate the mitotic progression. Intriguing results from Lara-Gonzalez et al. (2021b) showed depletion of BubR1 increased Mad2-GFP signals on kinetochores indicating BubR1 is able to release Mad2-Cdc20 from kinetochores by replacing Bub1 on binding to the β-propeller of Cdc20. As discussed in the Bub1-BubR1 section, the second pool of BubR1 is more dynamic and suitable to interact with newly formed Mad2-Cdc20 complex in close proximity than the Bub1-dependent pool.

### Bub1-RZZ Interaction may Contribute to Spindle Assembly Checkpoint Activation

Composed by Rod, Zwilch and ZW10, RZZ complex localizes on unattached kinetochores and provides another layer of SAC regulation in higher eukaryotic cells. So far, no RZZ homologs have been identified in yeast. Together with Spindly, an adaptor protein for dynein-dynactin, RZZ assembles and expands the outmost region of kinetochores to form a fibrous corona, which maximizes the chances of binding with microtubules. Meanwhile RZZ recruits Mad1-Mad2 to the outer kinetochores to monitor the interaction with microtubules (Basto et al., 2000; Chan et al., 2000; Buffin et al., 2005; Kops et al., 2005; Gama et al., 2017; Mosalaganti et al., 2017; Pereira et al., 2018; Rodriguez-Rodriguez et al., 2018; Sacristan et al., 2018; Zhang et al., 2019; Raisch et al., 2021; Barbosa et al., 2022). Once kinetochores are attached to microtubules, RZZ and Mad1-Mad2 are transported away from kinetochores *via* the Spindly/dynein/dynactin to silence SAC (Barisic et al., 2010; Gama et al., 2017; Gassmann et al., 2010; Pereira et al., 2018; Sacristan et al., 2018; reviewed in McHugh and Welburn, 2017).

How RZZ localizes onto kinetochores is not fully understood and currently direct protein interactions responsible for this is missing. Zwint, the chaperon protein of KNL1 was originally proposed as the kinetochore receptor for RZZ (Wang et al., 2004; Kops et al., 2005; Famulski et al., 2008; Vos et al., 2011). Later it was realized that the dependency is indirect due to the instability of KNL1 in the absence of Zwint (Zhang et al., 2015; van Hooff et al., 2017). Quantification of RZZ kinetochore signals in cells complemented with different KNL1 mutants revealed a requirement for Bub1-MELT interactions for efficient RZZ kinetochore localization. Further truncation analysis of Bub1 identified a region encompassing CD1 domain required for the robust RZZ kinetochore localization. Since efficient Bub1 depletion by RNAi could only remove 65%–85% of ZW10 from kinetochores, there are obviously other receptors for RZZ recruitment (Caldas et al., 2015; Zhang et al., 2015). In *Caenorhabditis elegans*, the unstructured N-terminal tail of Ndc80 interacts with Rod (Cheerambathur et al., 2013). Whether this interaction is conserved in mammalian cells and anchors RZZ on kinetochores needs to be characterized.

As discussed in Bub1-Mad1 section, the Bub1 CD1 domain interacts with the Mad1 C-terminal domain through multiple contacts. Is it Mad1 recruited by Bub1 that is responsible for RZZ kinetochore localization? Single amino acid mutation disrupting the CD1-Mad1 interaction did not reduce RZZ kinetochore signals which argues against Mad1 playing a direct role in RZZ recruitment (Zhang et al., 2019). The molecular mechanism of Bub1 promoting RZZ kinetochore localization needs further investigation.

Similar as Bub1, depletion of Rod removed around 50% of Mad1/Mad2 from kinetochores in cells treated with nocodazole. In either case, the SAC was significantly impaired but not completely abolished (Caldas et al., 2015; Silió et al., 2015; Rodriguez-Rodriguez et al., 2018; Zhang et al., 2019). When both Rod and Bub1 were depleted, Mad1 could not localize onto kinetochores anymore and the checkpoint was completely inactivated. Interestingly, tethering Mad1 onto kinetochores by fusing it to kinetochore protein Mis12 or KNL1 could fully restore the checkpoint defect in the absence of RZZ, but not in the absence of Bub1. This clearly shows distinct roles of RZZ and Bub1 in the SAC signaling (Rodriguez-Rodriguez et al., 2018; Zhang et al., 2019). Based on these observations, it is proposed that RZZ recruitment of Mad1 facilitates the interaction between Bub1 and Mad1 which catalyzes the production of MCC (Zhang et al., 2019; Figure 3). How Mad1 interacts with RZZ is not known yet. A recent study identified a region on Mad1 to be involved in interacting with RZZ has shed light on this question (Herman et al., 2021). The mechanism of RZZ-Mad1 facilitating Bub1-Mad1 interaction is also missing. The finding that the CD1 C-terminal end and the downstream region of Bub1 promote RZZ kinetochore localization may be part of the mechanism.

### Bub1-Cdc20 Plays Important Role on Spindle Assembly Checkpoint Activation

Cdc20 is a coactivator of APC/C as well as a component of MCC. It contains a disordered N-terminal region and a C-terminal β-propeller composed by seven WD40 repeats. Using bioinformatic and biochemical tools, a Cdc20 binding motif was identified in several proteins including cyclin A, BubR1, Bub1 and Acm1. The consensus of the motif is Fx [ILV] [FHY] × [DE] and was named as ABBA motif or Phe box (Di Fiore et al., 2015; Diaz-Martinez et al., 2015). The ABBA motif on Bub1 locates exactly after the region required for Mad1 and RZZ kinetochore localization (Figure 1). Functional analysis found the motif is important for checkpoint activation, though less critical as CD1 domain (Di Fiore et al., 2015; Zhang et al., 2017; Roy et al., 2022). As discussed in Bub1-Mad1 section, Bub1 positions Cdc20 close to Mad1-Mad2 and favors the formation of Mad2-Cdc20 (Figure 3). Interestingly, the kinetochore recruitment of Cdc20 by Bub1 ABBA motif may delay anaphase onset by other mechanism beyond MCC formation. There are evidences that Bub1, together with the bound kinase Plk1 is able to phosphorylate Cdc20 associated with Bub1 ABBA motif. The phosphorylated Cdc20 then binds and inhibits APC/C by preventing its interaction with the E2 ubiquitin-conjugating enzyme Ube2S (Tang et al., 2004; Craney et al., 2016; Jia et al., 2016). It is worth noting that Bub1-Cdc20 interaction may also accelerate APC/C activation under different circumstances. For example, in *C. elegans* embryos, once kinetochore-microtubule attachment is generated, protein phosphatase one binds to KNL1 and eliminates the phosphorylation on Cdc20 bound with Bub1. Unphosphorylated Cdc20 then works as an activator of APC/C to promote the metaphase-anaphase transition (Kim et al., 2017). Interestingly, in mammalian cells, both PP1 and PP2A-B56 were reported to dephosphorylate Cdc20 (Bancroft et al., 2020; Hein et al., 2021).

### Bub1-Plk1 Interaction Plays Minor Roles on Spindle Assembly Checkpoint Activation

Plk1 is an important mitotic kinase playing multiple roles on mitotic entry, centrosome maturation, bipolar spindle assembly, chromatid cohesioin, kinetochore-microtubule attachment and mitotic exit (reviewed in Combes et al., 2017). Plk1 binds to Bub1 through the C-terminal Polo-box domain (PBD) and the PBD binding motif on Bub1 (Qi et al., 2006; Ikeda and Tanaka, 2017). Emerging evidences indicate that Plk1 is involved in activating and maintaining the SAC signaling. When Plk1 alone gets inhibited, there is very limited effect on the checkpoint. The effect on checkpoint only becomes pronounced when Mps1 or Aurora B is partially inhibited simultaneously with Plk1 inhibition (Espeut et al., 2015; O’Connor et al., 2015; von Schubert et al., 2015; Ikeda and Tanaka, 2017). Two mechanisms have been proposed based on these evidences. The first one bypasses the MCC formation. Plk1 is able to phosphorylate Cdc20 on multiple sites, and the phosphorylated Cdc20 inhibits APC/C directly as discussed in Bub1-Cdc20 section (Craney et al., 2016; Jia et al., 2016). The second mechanism is through promoting the MCC formation. Plk1 phosphorylates Mps1 for its full activation as well as KNL1 on the MELT motifs to promote the kinetochore localization of checkpoint proteins and subsequent MCC production (Espeut et al., 2015; O’Connor et al., 2015; von Schubert et al., 2015; Ikeda and Tanaka, 2017; Cordeiro et al., 2020).

### Bub Kinase Plays Minor Roles on Spindle Assembly Checkpoint Activation

Bub1 is a bona fide kinase with a few substrates identified (Roberts et al., 1994; Tang et al., 2004; Kawashima et al., 2010; Asghar et al., 2015). The most characterized substrate is histone H2A. Phosphorylation of H2A on T120 provides centromere docking site for TOP2A and Sgo1, the latter further recruits Aurora B and PP2A to promote an accurate chromosome segregation (Kitajima et al., 2005; Tang et al., 2006; Boyarchuk et al., 2007; Fernius et al., 2007; Kawashima et al., 2010; Tsukahara et al., 2010; Broad et al., 2020; Hadders et al., 2020; Zhang et al., 2020). Whether the kinase activity is required for SAC is highly debated (Roberts et al., 1994; Sharp-Baker and Chen, 2001; Warren et al., 2002; Yamaguchi et al., 2003; Chen et al., 2004; Tang et al., 2004; Vanoosthuyse et al., 2004; Fernius et al., 2007; Klebig et al., 2009; Kawashima et al., 2010; Perera et al., 2010; Ricke et al., 2012; Baron et al., 2016; Faesen et al., 2017; Siemeister et al., 2018; Roy et al., 2019; Hadders et al., 2020). Careful examination of the above studies indeed revealed a limited role of the kinase on activating the checkpoint which might be through multiple mechanisms. By phosphorylating the N-terminal region of Cdc20, Bub1 could inhibit APC/C beyond the formation of MCC (Tang et al., 2004). By phosphorylating H2A T120, Bub1 promotes the centromere enrichment of Aurora B which further activates other players like Mps1 and Plk1 to fully activate the checkpoint (Saurin et al., 2011; Carmena et al., 2012; Shao et al., 2015; Singh et al., 2021). The third possibility is the auto-phosphorylation which may regulate interactions with other checkpoint proteins and kinetochore kinetics of Bub1 (Asghar et al., 2015).

## Bub1 in Chromosome Alignment

After nuclear envelope breaks down, the dispersed chromosomes need to attach to the microtubules and migrate to the cell center to form the metaphase plate. Aligning chromosomes at the center favors the accurate chromatids segregation. Disturbance of the congression or the failure to maintain the alignment will cause chromosome aligning defects and chromatids segregation errors. The mechanisms behind chromosome congression has been comprehensively reviewed (Maiato et al., 2017).

The role of Bub1 in supporting chromosome alignment was first observed almost two decades ago (Johnson et al., 2004; Meraldi and Sorger 2005; Perera et al., 2007), but the mechanism only starts to emerge recently. In the following, we discuss the known and the possible roles of the Bub1 interactors as well as the kinase activity on promoting chromosome alignment.

### Bub1-BubR1 Interaction Is Important for Chromosome Alignment

**FIGURE 4:**
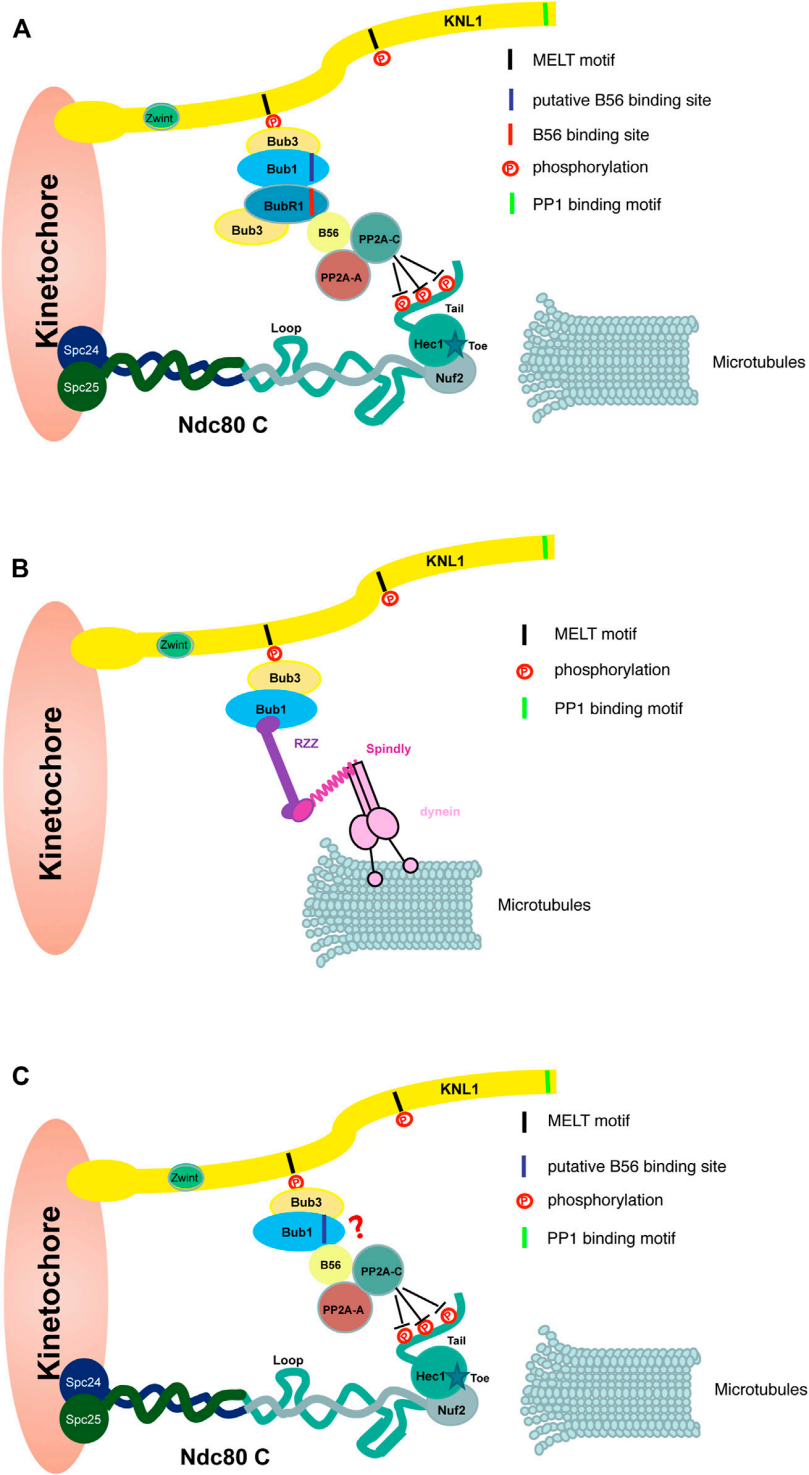
Bub1 promotes kinetochore-microtubule attachment through multiple possible pathways. The cartoon shows Bub1 promotes kinetochore-microtubule attachment through a few possible pathways. **(A)** Bub1 interacts BubR1 directly and the latter brings protein phosphatase PP2A-B56 onto outer kinetochores. The PP2A-B56 complex dephosphorylates multiple outer kinetochore proteins like Ndc80 shown here, which stabilizes the attachment between kinetochores and microtubules. **(B)** Bub1 promotes the kinetochore localization of RZZ complex. Through an interaction with dynein complex mediated by Spindly protein, Bub1-RZZ may facilitate the initial lateral interaction between kinetochores and microtubules. RZZ-Spindly is able to oligomerize into a fibrous corona which is not shown here. **(C)** Bub1 may interact PP2A/B56 directly through the putative B56 binding motif. PP2A/B56 promotes a stable kinetochore-microtubule attachment as described in **(A)** The contribution of the kinase activity is not shown here.

The mechanism of BubR1 in supporting chromosome alignment has been extensively studied. In summary, through direct interaction of a conserved LxxIxE motif on BubR1 with a surface-exposed pocket on B56, BubR1 recruits protein phosphatase PP2A-B56 onto kinetochores to antagonize the phosphorylation of outer kinetochore proteins like Ndc80, Dsn1, KNL1 and Ska3 (Suijkerbuijk et al., 2012; Kruse et a., 2013; Xu et al., 2013; Espert et al., 2014; Hertz et al., 2016; Wang et al., 2016; Maciejowski et al., 2017). Reducing the phosphorylation level of outer kinetochore proteins promotes stable attachment of kinetochores with microtubules. There are a few studies showing Bub1-dependent BubR1 is required for efficient chromosome alignment during mitotic progression (Zhang et al., 2016; Carvalhal et al., 2022; Figure 4A). Whether Bub1-BubR1 interaction plays the major role of Bub1 to promote chromosome alignment needs further investigation.

### Bub1-RZZ may Contribute to Chromosome Alignment

As mentioned in the above sections, Bub1 promotes RZZ complex localization onto unattached kinetochores (Caldas et al., 2015; Zhang et al., 2015). RZZ complex plays multiple roles to facilitate the kinetochore-microtubule attachment. For example, RZZ recruits Spindly and dynein-dynactin complex onto kinetochores (Gassmann et al., 2008; Barisic et al., 2010; Gama et al., 2017; Mosalaganti et al., 2017; Sacristan et al., 2018). Dynein-dynactin, the minus-end directed motor, facilitates the initial lateral kinetochore-microtubule attachment and a fast poleward movement of chromosomes. This is important for chromosomes, especially the ones close to the spindle poles to complete the congression (Li et al., 2007; Yang et al., 2007; Vorozhko et al., 2008). Together with Spindly, RZZ oligomerizes into a fibrous meshwork which drives the formation of the corona on outmost region of kinetochores (Pereira et al., 2018; Sacristan et al., 2018). The fibrous corona is able to facilitate the kinetochores-microtubules attachment, reduce merotelic attachment and promote spindle assembly (reviewed in Kops and Gassmann, 2020; Barbosa et al., 2022). How much does Bub1-dependent RZZ contribute to the process is less known. One recent study in *C. elegans* embryos demonstrated that Bub1 indeed regulates chromosome congression *via* recruiting RZZ/Spindly/dynein/dynactin onto kinetochores (Edwards et al., 2018; Figure 4B). Whether this is also the case in mammalian cells awaits further studies.

### Bub1-B56 Interaction Awaits Further Examination

Similar to its paralog BubR1, Bub1 also harbors a putative B56 binding motif (FSPIQE) at the similar position. An *in vitro* peptide binding assay suggests Bub1 may not bind B56 as efficiently as BubR1 (Braga et al., 2020). In contrast, the study in *C. elegans* oocytes found Bub1 was able to recruit PP2A/B56 via the B56 binding motif and the Bub1-B56 interaction was important for chromosome congression in meiosis I (Borja et al., 2020; Figure 4C). Again, a functional analysis in mammalian cells is needed to examine whether this motif is involved in promoting the chromosome alignment in mitosis.

### Bub1-Plk1 Interaction may be Dispensable for Chromosome Alignment

Plk1 kinase plays essential roles during mitosis including promoting chromosome alignment (reviewed in Combes et al., 2017). Whether the Bub1-Plk1 interaction is needed for chromosome alignment is not clear till recently. Three studies have identified Bub1 and CENP-U as the major kinetochore receptors for Plk1. Simultaneously blocking the two receptors caused strong chromosome aligning defects while disrupting either one alone did not (Chen et al., 2021; Nguyen et al., 2021; Singh et al., 2021). Interestingly, BubR1 also binds Plk1 which further phosphorylates BubR1 to enhance the binding with B56 (Elowe et al., 2007; Suijkerbuijk et al., 2012; Wang et al., 2016). Probably due to the negative feedback from PP2A/B56, BubR1 contributes less on Plk1 kinetochore localization than Bub1 or CENP-U (Singh et al., 2021). Different to Bub1 and CENP-U, disrupting the Plk1 docking on BubR1 severely impairs chromosome alignment (Elowe et al., 2007). Very likely, the Plk1 bound to Bub1 and CENP-U has different targets other than BubR1 to regulate the interaction between microtubules-kinetochores. It will be interesting to identify the substrates in future study.

### Bub1 Kinase Plays Minor Roles on Chromosome Alignment

Similar as in SAC signaling, the role of Bub1 kinase on the chromosome alignment is also highly inconsistent (Warren et al., 2002; Fernius et al., 2007; Klebig et al., 2009; Kawashima et al., 2010; Perera et al., 2010; Ricke et al., 2012; Baron et al., 2016; Siemeister et al., 2018; Broad et al., 2020; Hadders et al., 2020; Liang et al., 2020; Zhang et al., 2020; Chen et al., 2021; Carvalhal et al., 2022). For example, the kinase dead Bub1 could not rescue the chromosome alignment defects caused by depleting endogenous Bub1 in HeLa and RPE cells (Klebig et al., 2009). This indicates the kinase activity is a key regulator for chromosome alignment. In contrast, HeLa cells with Bub1 kinase inactivated by CRISPR/Cas9 only displays mild mitotic defects (Chen et al., 2021). The mitosis took 30 min longer than control cells due to the delayed chromosome alignment. Eventually all chromosomes aligned on the metaphase plate and segregated correctly. It has to be noted that in these cells, the Bub1 protein was expressed at low levels (3%–10% to parental cells). The delayed chromosome alignment may be caused by both low Bub1 protein level and kinase inactivation. In this case, the mild mitotic defects does not support an important role for the kinase activity on chromosome alignment.

The discrepancy also exists in the studies with mouse embryonic fibroblasts cells. Perera et al. (2010) found kinase dead Bub1 could fully restore the chromosome alignment and segregation defects in mouse embryo fibroblasts (MEFs) when *BUB1* was conditionally inactivated. Another study of *Bub1* ^*KD/KD*^ (KD means kinase dead) MEFs found a significant increase of rates with misaligned chromosomes at the onset of anaphase compared with wild type cells. However, there were still around 80% of *Bub1* ^*KD/KD*^ MEFs without chromosome alignment defects or mitotic delay (Ricke et al., 2012). Compared with the Bub1 knockout MEFs, the alignment defect is much milder in *Bub1* ^*KD/KD*^ MEFs indicating the kinase activity of Bub1 may contribute to the chromosome alignment, but not as the main driving force. Consistently, *Bub1* ^*KD/KD*^ mice were viable while *Bub1* ^Δ*/*Δ^ died after day E3.5 (Perera et al., 2007).

Recently, the first two patients with biallelic germline *BUB1* mutations were reported (Carvalhal et al., 2022). One of the patients harbors a short deletion in the kinase domain. In fibroblast cells derived from the patient, the protein level of the kinase-inactive Bub1 was significantly reduced and the mitotic process was 24 min delayed with a high frequency of segregation errors in anaphase. Again, the phenotype could be a combined result of both low protein level and kinase inactivation. Besides the genetic inactivation, a series of studies using chemical inhibition of the Bub1 kinase activity revealed either none or marginal mitotic defects (Baron et al., 2016; Siemeister et al., 2018; Broad et al., 2020; Hadders et al., 2020; Liang et al., 2020; Zhang et al., 2020).

In general, majority evidences do not favor an important role of Bub1 kinase activity in promoting chromosome alignment. The minor role of Bub1 kinase may play in this function could be through recruiting Aurora B onto centromeres since artificial centromeric tethering of INCENP, an interactor of Aurora B, could fully rescue the chromosome misalignment in *Bub1* ^*KD/KD*^ MEFs (Ricke et al., 2012). The mechanism of the rescue is not clear yet. The prevailing model proposes the centromere localized Aurora B phosphorylates outer kinetochore proteins to destabilize and correct erroneous attachment (Liu et al., 2009; Lampson and Cheeseman, 2011). Therefore, inactivation of Bub1 kinase results in Aurora B delocalization and impairs its ability to correct attachment errors. In contrast to this model, the phosphorylation levels of outer kinetochore proteins like Hec1 pS44 were not affected or only moderately reduced (25%–40%) in the cells without centromere Aurora B after simultaneous inhibition of both Bub1 and Haspin (Hadders et al., 2020; Broad et al., 2020; Liang et al., 2020). Since most of the phosphorylation remains on kinetochores, a kinetochore pool of Aurora B has been suggested responsible for outer kinetochore protein phosphorylation. Interestingly, Bub1 itself has been proposed as one kinetochore receptor for Aurora B which needs to be verified in the future (Broad et al., 2020).

Another possibility is the full activation of Plk1 requires centromeric Aurora B (Carmena et al., 2012; Shao et al., 2015; Singh et al., 2021). Plk1 plays important roles on the kinetochore-microtubule attachment as discussed in Bub1-Plk1 section. Thus, delocalized Aurora B reduces the kinase activity of Plk1 when Bub1 kinase is under inhibition. Less active Plk1 may not efficiently phosphorylate BubR1 or other substrates to promote the stable kinetochore-microtubule attachment.

One interesting observation from the patient cells without Bub1 kinase activity is the chromatid bridges with centromeres separated which indicates errors on chromatid arms instead of on kinetochores. Inactivation of Bub1 kinase activity causes delocalization of Sgo1/PP2A from centromeres to the chromosome arms which may protect cohesin complex on chromosome arms from being removed by Plk1 and Aurora B (Kitajima et al., 2005; Tang et al., 2006; Fernius et al., 2007; Kawashima et al., 2010; Carvalhal et al., 2022). Bub1 kinase is also required for TOP2A centromere enrichment. Delocalized TOP2A can not efficiently resolve sister chromatid entanglements which results in ultra-fine bridges (UFBs) during anaphase (Zhang et al., 2020).

### Bub1-CENP-F Interaction may be Dispensible for Chromosome Alignment

CENP-F is a long coiled-coil protein containing two microtubule binding domains. In mitosis, CENP-F localizes on kinetochores through direct interaction with the C-terminus of Bub1 (Rattner et al., 1993; Liao et al., 1995; Johnson et al., 2004; Feng et al., 2006; Musinipally et al., 2013; Volkov et al., 2015; Ciossani et al., 2018; Raaijmakers et al., 2018). Furthermore, CENP-F recruits dynein through Nde1/Ndel1/Lis1 complex (Stehman, et al., 2007; Vergnolle et al., 2007; Wynne et al., 2018). Early studies discovered important roles of CENP-F on chromosome alignment by RNA interference (Bomont et al., 2005; Holt et al., 2005; Yang et al., 2005; Feng et al., 2006). The work in *C. elegans* embryos also found Bub1 contributes to the kinetochore-microtubule attachment *via* CENP-F/CLASP axis (Edwards et al., 2018). However, recent studies by knocking out *CENP-F* showed different results. First, *CENP-F* knockout mice were viable which questions the essentiality of the gene (Haley et al., 2019). Second, the *CENP-F* knockout cell lines displayed none mitotic phenotype or very mild effect on mitotic progression (Raaijmakers et al., 2018; Auckland et al., 2020). Though lack of enough evidences, an important role of Bub1-CENP-F interaction on chromosome alignment is disfavored in mammalian cells.

## Bub1 Essentiality

Considering the critical roles Bub1 plays in mitosis as discussed above, it is hardly to question its essentiality in higher eukaryotes. Indeed, in mouse studies, knockout of *BUB1* resulted in embryonic lethality after day E3.5. Late inactivation on day E10.5 quickly shut down further development. In MEFs cells, knocking out of Bub1 severely impaired SAC and chromosome alignment and the cell proliferation stopped due to the strong chromosome segregation errors (Perera et al., 2007; Tilston et al., 2009). *BUB*1 hypomorphic mice expressing minimal level of Bub1 truncate (less than 5% to wild type mice) were viable with increased tumorigenesis (Schliekelman et al., 2009). In humans, low Bub1 expression correlates with spontaneous miscarriage (Shi et al., 2011). Recently, two patients with biallelic germline *BUB1* mutations were reported. Patient one has homozygous point mutation in the start codon of *BUB1* resulting in residual levels of Bub1 protein beyong detection by regular methods. The existence of H2A pT120 signals on kinetochores indicates the presence of a functional Bub1 protein in this patient. The other patient expresses a low level of truncated Bub1 protein without kinase activity. Both patients suffered microcephaly, delayed development and other abnormalities (Carvalhal et al., 2022).

The above studies clearly show *BUB1* is essential in higher eukaryotes. However, the successful generation of *BUB1* knockout human cells seriously questioned the essentiality (Currie et al., 2018; Raaijmakers et al., 2018; Rodriguez- Rodriguez et al., 2018; Zhang et al., 2019; Chen et al., 2021). Further studies found all these *BUB1* knockout cell lines still expressed low level of Bub1 truncates by nonsense-associated alternative splicing (Rodriguez- Rodriguez et al., 2018; Zhang et al., 2019; Chen et al., 2021). The residual Bub1 (3%–30% of parental cells) was able to fully support the SAC and largely promote chromosome alignment in the cells. Thus, the mitotic progression was only moderately elongated with one or two chromosomes delayed on joining the metaphase plate. The functional checkpoint ensured the delayed chromosomes could align properly before cells entered anaphase without severe chromosome segregation errors. One interesting feature of these cells is the super sensitivity to RNA interference. Treatment of the cells with siRNA oligos against Bub1 resulted in severe impairment on both SAC signaling and chromosome alignment and cells entered anaphase with massive unaligned chromosomes which was hardly achieved in normal cells (Zhang et al., 2019; Chen et al., 2021). Importantly, supplemented with siRNA resistant wild type Bub1 fully rescued these defects indicating Bub1 is indeed required for faithful mitosis (Zhang et al., 2019). So far, by introducing indels in either exons for N-terminal region or exons for C-terminal region failed to completely inactivate Bub1. The effort to generate a full deletion of *BUB1* in diploid or aneuploid human cells also failed. The failure could be from the technology difficulty or from the possibility that diploid or aneuploid cells can not survive the full *BUB1* deletion. Only in near-haploid HAP1 cells, a full *BUB1* deletion by simultaneously targeting exon 1 and exon 25 was achieved showing *BUB1* is not essential in HAP1 cells. In these cells, the SAC strength was reduced at least in the presence of low concentration of microtubule toxin (Raaijmakers and Medema, 2019). It needs to be noted that having only one set of genome may confer different response to the loss of essential genes in HAP1 cells than in diploid or aneuploid cells.

**FIGURE 5:**
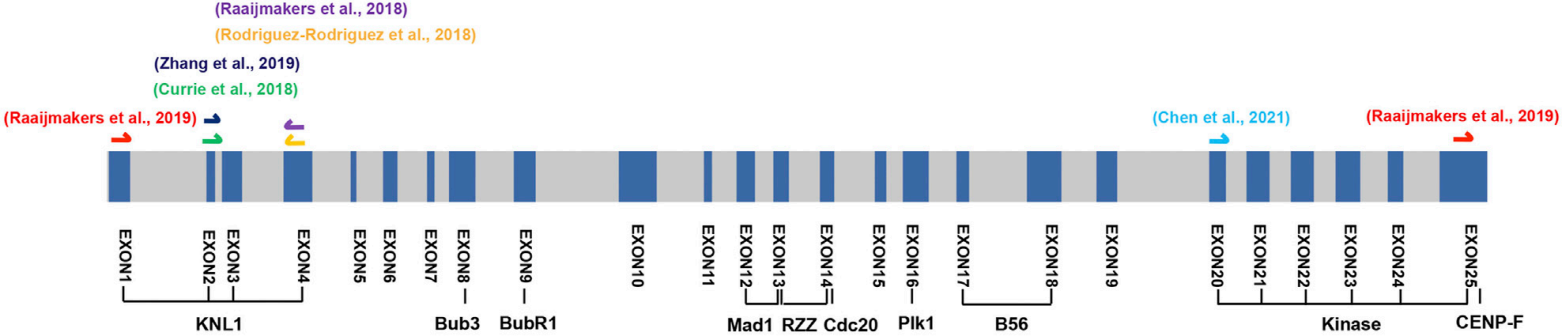
Schematic of the targeted exons of the Bub1 knockout cell lines. The schematic shows all the exons of BUB1 in human genome. The exons targeted by CRISPR/Cas9 from a number of studies are marked on top of the schematic. We propose targeting exon 8 (GLEBS domain) or co-targeting exon 9 (BubR1 binding domain) and exon 12 (CD1 domain) may cause lethality. The lengths of exons, but not introns are proportional to the actual lengths on genome.

An alternative strategy to confirm the essentiality of *BUB1* in human cells is introducing indels in the exons encoding domains with important functions (McKinley and Cheeseman, 2017). Alternative splicing normally escapes part or the whole exon Cas9 targeted. Thus, the truncated proteins if possibly produced, are not able to execute these functions. Examining the viability of the cells following the Cas9 cutting will provide direct answer on the essentiality of *BUB1*. Exons 8, 9, and 12 encoding Bub3, BubR1, Mad1 binding domains are particularly interesting for this purpose. Disrupting exon 8 will prevent Bub1 truncate from kinetochores which is very likely lethal as Perera et al. (2010) showed in MEFs cells. Singly cutting exon 9 or 12 will impair the chromosome congression or SAC signaling which may not be enough to cause lethality due to the protection by full or partial functional checkpoint. Simultaneously targeting exon 9 and 12 is more likely to be intolerable for cells (Figure 5).

In general, after 30 years’ extensive investigation, the importance of Bub1 in mitosis has been well documented and largely appreciated with some questions undissolved yet. On the checkpoint: how does Bub1 promote RZZ kinetochore localization? what is the molecular mechanism that RZZ-Mad1 facilitates Bub1-Mad1 interaction? On chromosome alignment: are there more domains of Bub1 required for chromosome alignment? what is the role of the putative B56 binding motif on Bub1? are the two B56 binding motifs on Bub1 and BubR1 redundant? On the essentiality: can human cells tolerate the complete loss of Bub1? Answer these questions will help us further understand the roles of Bub1 in mitosis.
